# The Downregulated Lipo-Related Gene Expression Pattern in Keloid Indicates Fat Graft Is a Potential Clinical Option for Keloid

**DOI:** 10.3389/fmed.2022.846895

**Published:** 2022-05-23

**Authors:** Xueqing Li, Zhaowei Zhu, Yangbin Xu, Shuqia Xu

**Affiliations:** Department of Plastic Surgery, The First Affiliated Hospital of Sun Yat-Sen University, Guangzhou, China

**Keywords:** keloid, fibroblast, fat grafting, pre-adipocyte, Gene Expression Omnibus

## Abstract

**Background:**

Keloids are a common complication of wounds, often manifesting with continuous hyperplasia and aggressive growth. Keloids also have a high recurrence rate and are largely resistant to treatment, making them clinically incurable, highlighting the need to translate basic research into clinical practice.

**Materials and Methods:**

We used GSE158395 and GSE92566 as discovery datasets to identify specific enriched hub genes and lncRNAs associated with keloid development and progression. This data was then used to identify the competing endogenous RNAs (ceRNAs) in these pathways by using a bidirectional selection method. Then, all hub genes and lncRNAs in ceRNAs were validated using GSE90051, GSE178562, and GSE175866, which describe the transcriptional profiles of keloid tissues, fibroblasts from pathological scars, and keloid fibroblast subpopulations, respectively. The keloid tissues were measured with qPCR.

**Results:**

Both fat-associated biological processes and fat cell differentiation were enriched in the downregulated gene set. Further evaluation revealed that all 11 hub genes were lipo-related, and most of these were differentially expressed in all three validation datasets. We then identified a clear ceRNA network within the data comprising six hub genes and four lncRNAs. Evaluations of the validation datasets confirmed that all six of these hub genes and two of the four lncRNAs were downregulated in keloid tissues; two hub genes and one lncRNA were downregulated in fibroblasts from pathological scars; and five hub genes and one lncRNA were significantly downregulated in mesenchymal subpopulation. Three genes had statistical difference and eight genes showed downregulated trend through qPCR of the keloid tissue.

**Conclusion:**

Our results suggest that keloid development relies on the downregulation of lipo-related genes and pre-adipocytes in diseased tissues and may be one of the key mechanisms underlying fat grafting-mediated treatment of pathological scarring.

## Introduction

Pathological scars, which are defined as abnormal products of wound repair involving the dermis, may occur in almost any patient (1). Keloids appear after trauma in susceptible individuals but may develop spontaneously with certain syndromes and chronic inflammatory skin conditions (2, 3). As a type of pathological scar, keloids demonstrate tumor-like behaviors (4), often causing itching, pain, and disfigurement; keloids can ulcerate with further trauma leading to chronic wounds that can potentially lead to high-risk cutaneous squamous cell carcinoma (1, 5). This is a result of the epithelial-to-mesenchymal transition (EMT) of keratinocytes, abnormal inflammation responses, and angiogenesis associated with such scars with these features being heavily reliant on diseased fibroblasts (6–8).

Current research into keloid scarring builds on the hypotheses surrounding initial scar tension and abnormal endocrine and immunological responses with most mainstream research focused on the immunocytology, epigenetics, and EMT of these tissues by using models developed from keloid-derived mesenchymal stem cells (9). However, research evaluations of fibroblast heterogeneity found that adult skin fibroblasts and keloid tissues are made up of at least four subpopulations including secretory-papillary, secretory-reticular, mesenchymal, and pro-inflammatory cells (10, 11). There are also a few other fibroblast subpopulations, such as the pericytes and pre-adipocytes, which are likely present in these tissues at very low levels (10, 12, 13). The mesenchymal fibroblast subpopulation is significantly increased in keloid tissues and closely associated with the overexpression of collagen in these tissues (11). Given the close relationship between these stem cells and skeletal development, ossification, and osteoblast differentiation, mesenchymal fibroblasts may intersect with keloid-derived mesenchymal stem cells, which retain the potential to differentiate into osteoblasts and chondrocytes (11, 14, 15).

Among the topical and intralesional treatments used for keloids, intralesional steroids seem the most effective. Other treatment options include surgical excision with modified closure and possible adjuvant radiation. However, patients may require multiple treatment sessions or develop recurrence at the same site or a different area (1). The data on mesenchymal stem cells have inspired the progress of clinical treatment, and many studies have focused on the use of these cells to treat and prevent pathological scars. Of the many treatments, fat transplantation, which relies on the effects of adipose-derived stem cells (ADSCs), has shown the most potential in these settings and has become an increasingly specific focus in clinical applications (16, 17).

Although the pathogenesis of keloids remains largely ill-defined and the mechanism underlying fat transplantation mediated repair of pathological scarring remains unknown, the increasing transcriptomic data for these conditions allows us to start to unravel these complexities. In this study, we used Gene Expression Omnibus (GEO) datasets to identify and validate changes in the transcription of lipo-related genes and cell components in keloid tissues, with this model not only explaining the pathogenesis of keloids but also providing a theoretical basis for the treatment of keloids using mesenchymal or fat cell transplantation therapies.

## Materials and Methods

### Dataset Acquisition and Processing

We used “keloid” as a keyword to identify relevant datasets in the GEO (http://www.ncbi.nlm.nih.gov/geo/) database. We then filtered this request further; identified five specific GEO datasets, GSE158395, GSE92566, GSE90051, GSE178562, and GSE175866, as suitable for our evaluations; and downloaded the relevant data. We used the first two as discovery datasets, and the others for validation. R (version 4.1.0) was used for all of the following procedures, and our figures were produced using the ggplot2 package (version 3.3.5).

GSE158395 comprised the Illumina NovaSeq 6000 expression profiling data for chronic keloid, emerging keloid, non-lesion skin biopsies (at least 10 cm away from a keloid), and their normal skin control (18). GSE92566 comprised the Affymetrix Human Genome U133 Plus 2.0 Array data and reports the expression profiling of chronic keloid, newly formed keloid, and adjacent non-lesion skin tissues (15). Three paired-groups from GSE178562 and GSE92566, including chronic keloid and adjacent non-lesion tissues, were selected for evaluation with all of these biopsies sampled from African American patients.

GSE90051 is based on Agilent-014850 Whole Human Genome Microarray 4 × 44K G4112F, which is a two-channel array containing keloid and adjacent normal tissue samples from seven Japanese patients (19). We also selected the fibroblasts of normal skin, normal scar, hypertrophic scar, and keloid data from GSE178562 for validation, with these data being produced by an Illumina HiSeq 2500 (13). GSE175866 is based on Illumina NovaSeq 6000 data and includes the transcriptional profile for CD266+/CD9- keloid fibroblasts (KZ), which are often also described as a subpopulation of mesenchymal fibroblasts and keloid fibroblasts except for the CD266+/CD9- ones (KF) (11).

Intersecting differentially expressed genes from the GSE158395 and GSE92566 datasets were then used as the target dataset. Differential expression was evaluated via a moderated paired *t*-test for multiple groups using the limma package for R (version 3.48.3) analysis (20), and the threshold values for significance were a |log2 fold change| of ≥1 and a *p*-value of < 0.05. Gene type information was then obtained from the relevant Genomic Data Commons (GDC) reference files (https://gdc.cancer.gov/).

### Gene Ontology (GO) Enrichment

Entrez IDs of each of the genes in the target dataset were then used to facilitate GO enrichment, and were obtained using the org.Hs.eg.db package for R (version 3.13.0) (21). The upregulated and downregulated genes were enriched separately using the clusterProfiler package for R (version 4.0.5) (22).

### Hub Gene Identification

Protein coding genes from the target dataset were inputted into STRING (version 11.5) (http://www.ncbi.nlm.nih.gov/geo/) and used to produce a protein-protein interaction (PPI) network. Hub genes were then selected using both CytoHubba (version 0.1) and MODE (version 2.0.0) in Cytoscape (version 3.8.2) (23–25). Expression patterns for each of the hub genes were obtained using the “HPA RNA-seq normal tissue” dataset from GEO.

### CeRNA Identification

The mirTarbase (version 7.0) of the MultiMiR package for R (version 2.3.0) was used for hub genes in the target dataset to identify the combinative miRNAs, including mRNA-miRNA validated by reporter assay, qRT-PCR, ChIP-seq, microarray, HITS-CLIP, western blot, and CLASH (26). The LncBase Experimental (version 2) was used for lncRNAs in the target dataset to obtain combinative miRNAs (27). The two groups of miRNAs were merged according to the miRNA to obtain the corresponding ceRNA network. The network figure was output using CytoHubba.

### Gene Expression Pattern Validation in Datasets

Hub genes, lncRNAs in the ceRNA network, and marker genes for each subpopulation of fibroblasts [*APCDD1* for secretory-papillary, *SLPI* for secretory-reticular, *POSTN* for mesenchymal, and *APOE* for pro-inflammatory cells (8)] were all used in the validation process. GSE90051 was used to validate the expression pattern of hub genes in keloids vs. adjacent normal tissues in different races from the discovery datasets, while GSE178562 was used to validate the expression pattern of hub genes in fibroblasts associated with keloids and other related tissues. GSE175866 was used to validate the expression patterns of hub genes in different keloid fibroblast subpopulations.

### Gene Expression Pattern Validation in Keloid Tissues

Real-time PCR was performed to detect changes in gene expression pattern in trunk keloids vs normal dermis and core of ear keloids vs their scar flaps. The clinical information is shown in Table S1.

## Results

### Identification and Enrichment of Differentially Expressed Genes From Keloid Tissues

**Figure 1:**
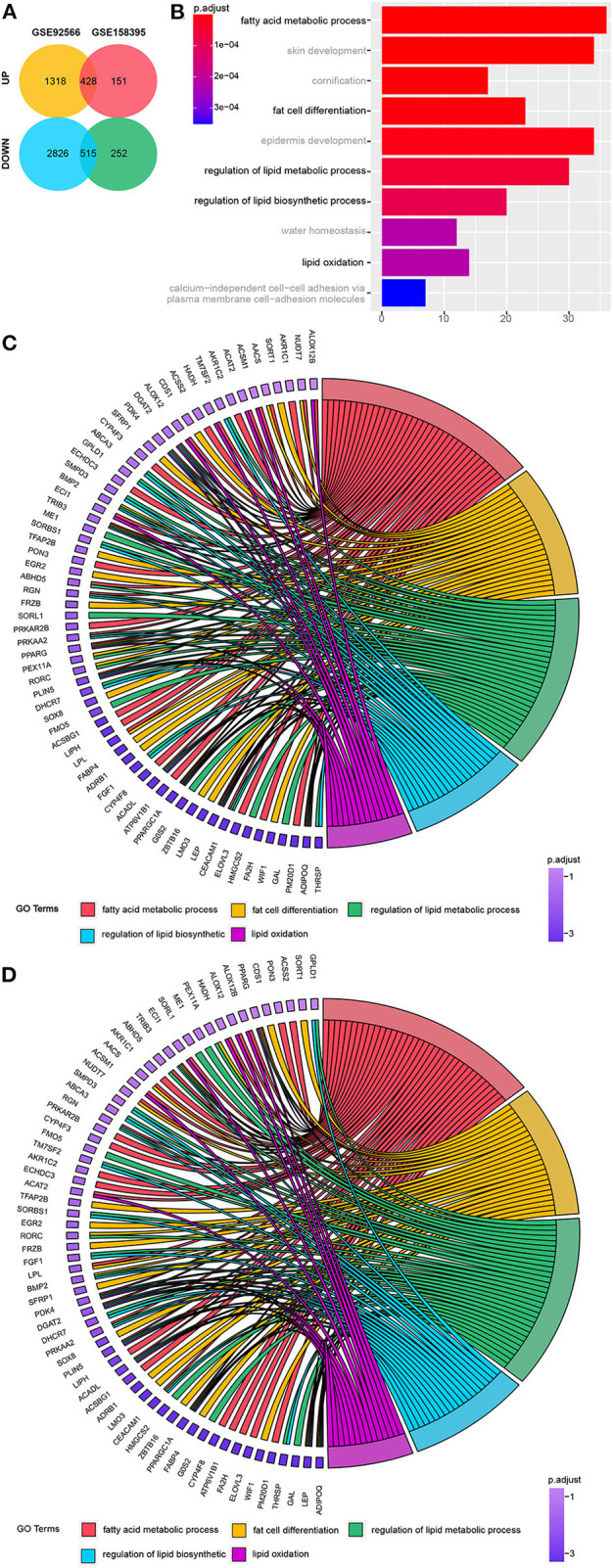
Identification of 943 differentially regulated genes. **(A)** Venn diagram of the differentially regulated genes in GSE92566 and GSE158395. **(B)** Top 10 Biological Process (BP) terms associated with the downregulated genes identified via Gene Ontology (GO) enrichment analysis. **(C, D)** Lipo-related genes from the top 10 lipo-related BP terms from GSE158395 and GSE92566.

Once the datasets had undergone their initial preprocessing and merger, we were left with a total of 428 upregulated and 515 downregulated genes for evaluation (Figure 1A), including 12 upregulated and 10 downregulated lncRNAs.

GO analysis revealed enrichment of chondrocytic and osteogenic genes in six of the top ten biological processes terms enriched in the upregulated gene dataset (Figure S1A). While five of the top 10 downregulated GO terms were linked to fat-associated biological processes and fat cell differentiation (Figure 1B). Clustering of cartilage- and bone-related and fat-related genes is shown in Figures S2A,B. The critical lipo-related genes identified here and their categories are shown in Figures 1C,D.

### Identification of Hub Genes

**Figure 2:**
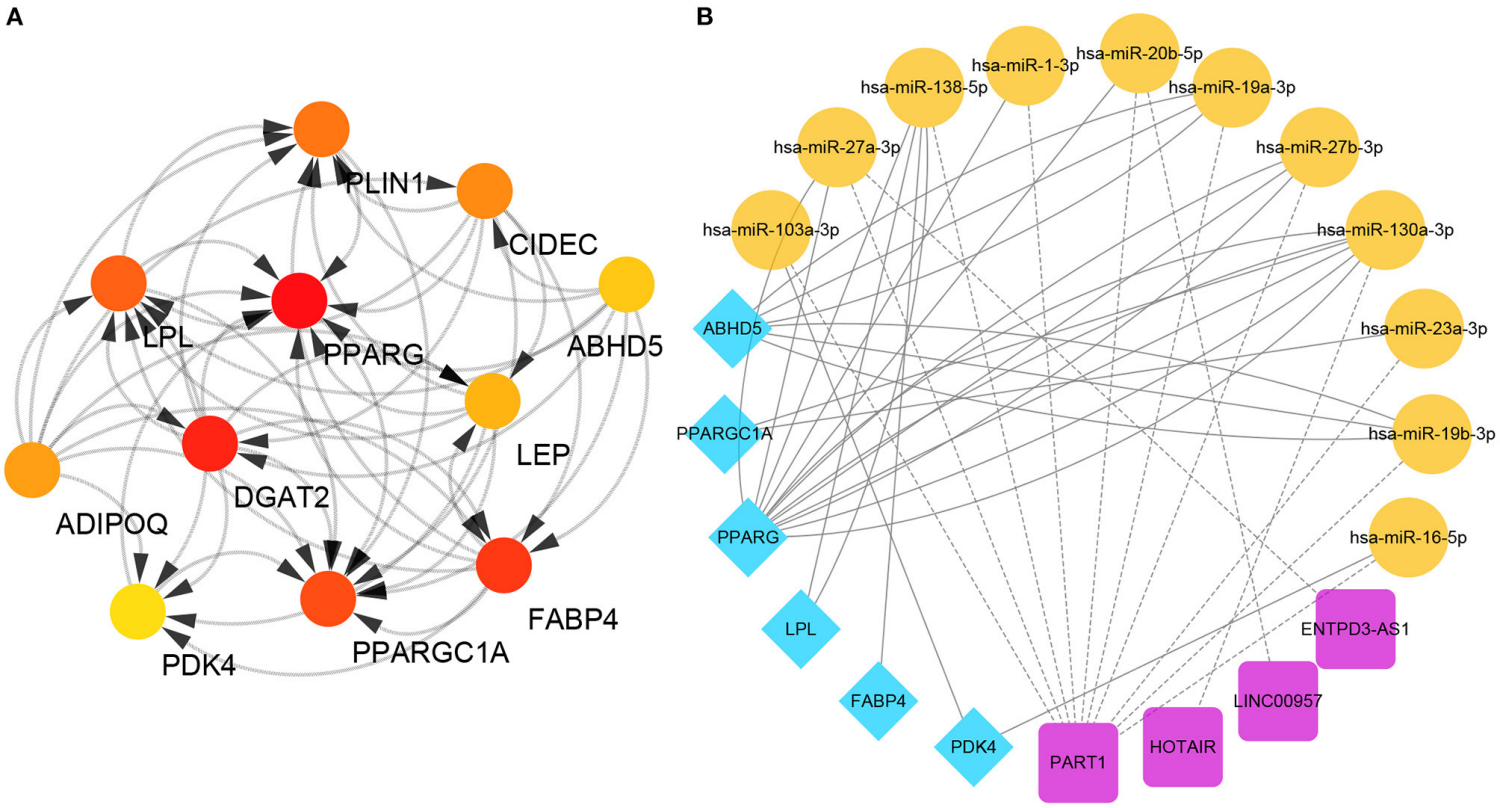
Identification of specific hub genes. **(A)** Protein-protein interaction (PPI) network. The redder the color, the higher the maximal clique centrality (MCC) score. **(B)** ceRNAs associated with each of the specific hub genes.

These downregulated genes were then used to identify the hub genes associated with the keloid phenotype and, although in a different order, both the MCODE prior cluster and the maximal clique centrality (MCC) evaluations identified the same top 11 genes based on the topology produced by CytoHubba (Figure 2A). These 11 genes were *LPL, PDK4, ABHD5, PLIN1, ADIPOQ, CIDEC, PPARG, PPARGC1A, LEP, FABP4*, and *DGAT2*. The correlation between these genes is shown in Figures S3A,B. Interestingly, these genes were all lipo-related, and with the exception of *PLIN1* and *CIDEC*, the others were all included in the top 10 lipo-related enrichment terms. This was further supported by the fact that these hub genes are typically highly expressed in fat tissues (Figure S4).

We then used bidirectional filtration to identify the ceRNAs for six of these hub genes, identifying four lncRNAs (PART1, HOTAIR, LINC00957, and ENTPD3-AS1) and 11 miRNAs (miR-103a-3p, miR-27a-3p, miR-138a-5p, miR-1-3p, miR-20b-5p, miR-19a-3p, miR-27b-3p, miR-130a-3p, miR-23a-3p, miR-19b-3p, and miR-16-5p) as critical members of this network (Figure 2B).

### Validation of Gene Expression Patterns in Different Keloid Tissues and Cells

Validation datasets confirmed the value and identity of the hub genes identified in our earlier evaluations in each step of the keloid process. First, in the keloid tissues, then in the fibroblasts of keloids, and finally in the keloid fibroblast subpopulation. However, variations in the platforms used to produce the transcriptional data did have some effect on the detection of some hub genes, but each of these genes could be annotated, regardless of statistical differences and are shown in their associated figures.

**Figure 3:**
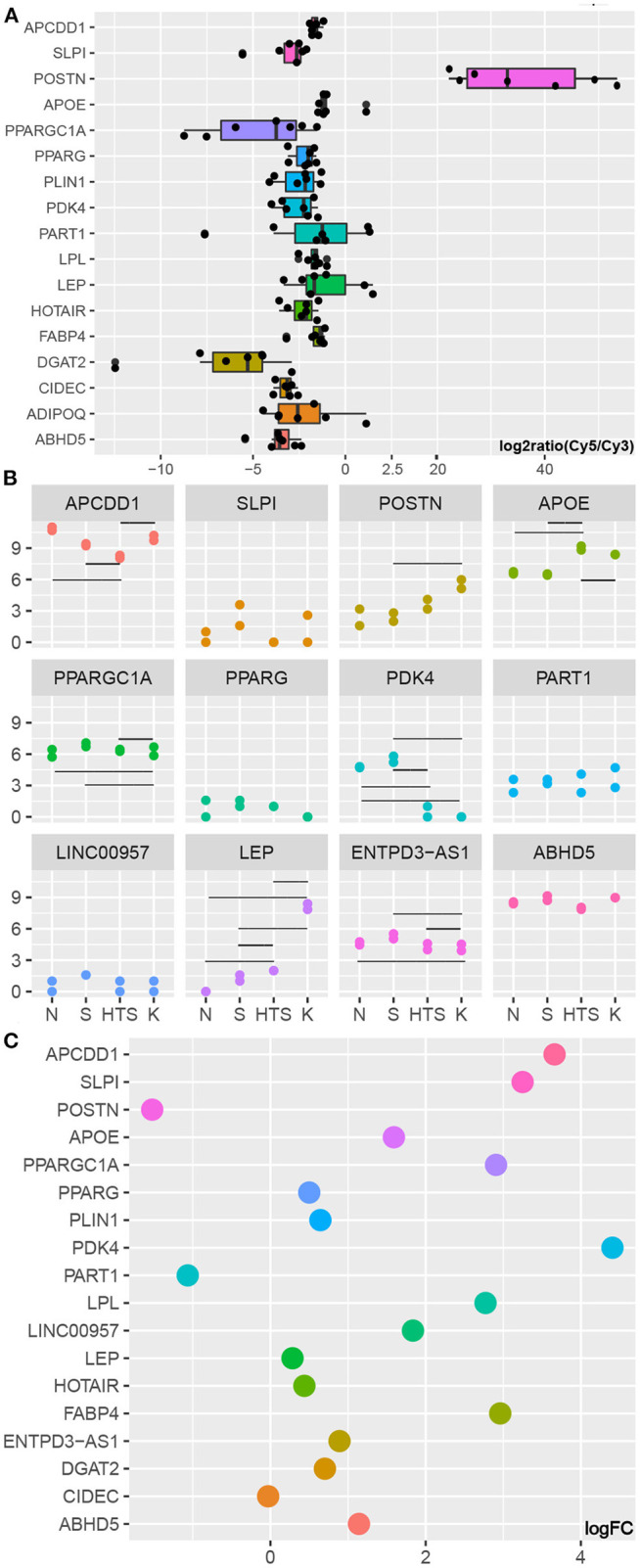
Validation of hub gene expression. **(A)** Marker and hub genes in GSE90051. **(B)** Marker and hub genes in GSE178562. Y represents log_2_(count+1) while the horizontal lines represent statistically significant differences between specific groups (Table S2). **(C)** Marker and hub genes in GSE175866. KF represents the control group.

GSE90051 described hub gene expression in keloid vs. adjacent non-lesion skin tissues and its evaluation confirmed the changes in the expression of the lipo-related hub genes *ABHD5, ADIPOQ, CIDEC, DGAT2, FABP4, LEP, LPL, PDK4, PLIN1, PPARG*, and *PPARGC1A*, as well as lncRNAs HOTAIR and PART1 in these tissues (Figure 3A).

GSE17856 includes data from a wide spectrum of fibroblasts from normal skin, normal scars, and pathological scars (hypertrophic and keloid) and revealed that the expression of *PDK4* is downregulated in the fibroblasts of pathological scars when compared with that in normal skin and normal scar fibroblasts. *PPARGC1A* expression was also lower in keloid fibroblasts than in the other three groups. However, the expression of *LEP* gradually increased as the degree of abnormality in the fibroblast spectrum increased. In addition, we were able to annotate ENTPD3-AS1, LINC00957, and PART1 in these data, and we revealed that lncRNA ENTPD3-AS1 was downregulated in keloid fibroblasts when compared to that in hypertrophic scars, normal scars, and normal skin, while there was no significant difference in the expression of any of the other lncRNAs in these four groups (Figure 3B; Table S2).

Figures 3A–C clearly show that *POSTN* is upregulated in keloid tissues, fibroblasts, and KZ fibroblasts, which is in agreement with the results of several recent studies showing that *POSTN* can be used as a marker of mesenchymal fibroblasts and that this mesenchymal fibroblast subpopulation increases in keloid tissues (11). In addition, Figure 3C shows that almost all of the genes of interest evaluated in this study, with the exception of *PART1*, were downregulated in KZ cells when compared to that in KF cells. We also found that with the log_2_ fold change threshold set to 1, the changes in *PPARGC1A, PDK4, LPL, FABP4, ABHD5*, and lncRNA LINC00957 were all shown to have statistical significance. Given the characterization of the adipogenic genes (12), we hypothesized that the 11 hub genes identified here represent the pre-adipocytes in the fibroblast subpopulation. When this is combined with the fact that the proportion of KF cells decrease in these samples and that evaluations of GSE90051 and GSE1788562 confirmed that these hub genes are likely to be downregulated in keloids, we can assume that this difference is the result of a reduction in the pre-adipocyte subpopulation (one part of KF cells) in keloid tissues.

### QPCR in Keloids of Trunk and Auricular

**Figure 4:**
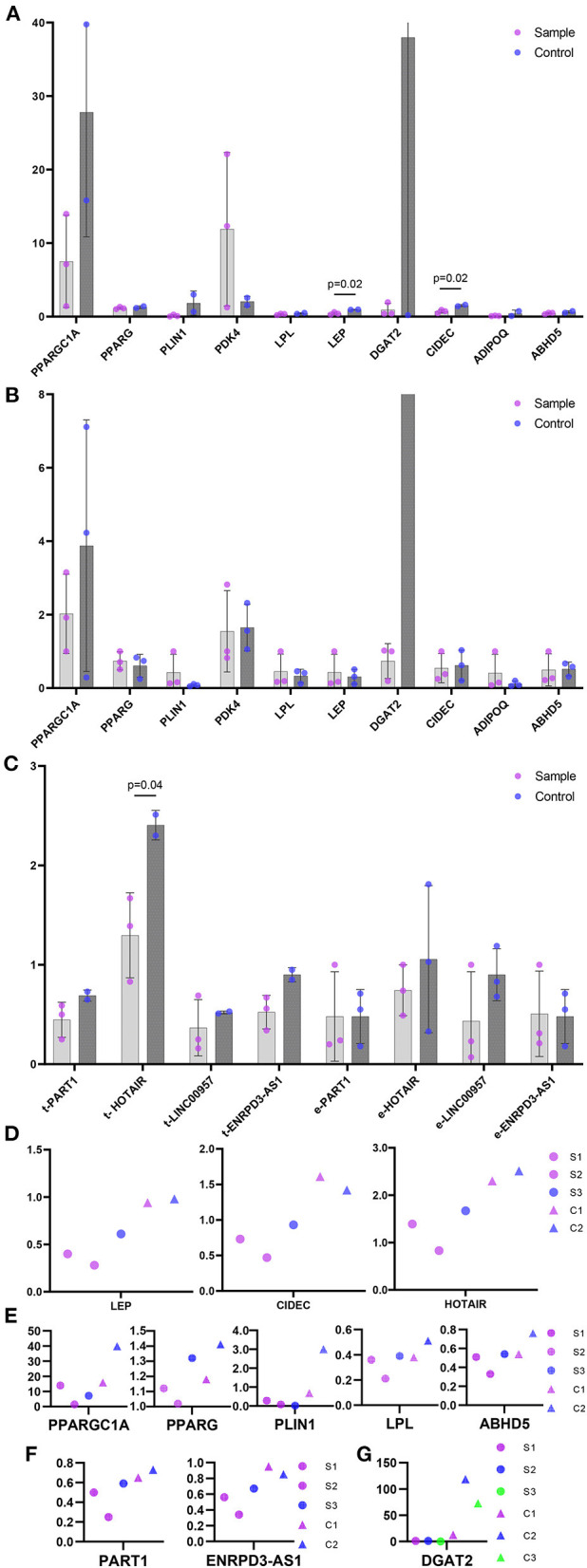
The relative expression level of hub genes and lncRNAs in ceRNAs. **(A)** Hub genes in trunk keloids vs normal dermis. Sample represent trunk keloid. Control represents normal dermis. **(B)** Hub genes in ear keloids vs their scar flaps. Sample represent ear keloid. Control represents their scar flaps. **(C)** lncRNAs. t- stands for trunk, and e- stands for ear. Sample represent keloid. Control represents their corresponding control groups. **(D)** Genes showed statistical difference. **(E-G)** Genes showed downregulated trend. **(E)** Hub genes in trunk keloid. **(F)** lncRNA in trunk keloid. S1, S2, S3 stand for trunk keloid 1, 2, 3 respectively. C1 and C2 stand for normal dermis of trunk 1 and 2. **(G)** lncRNA in ear keloid. S1/C1, S2/C2 and S3/C3 stand for ear keloid 1, 2, 3 and their scar flaps respectively.

PCR showed that LEP, CIDEC and lncRNA HOTAIR showed increased expression (*p* < 0.05) in trunk keloid of trunk, while none showed statistical difference in ear keloid (Figures 4A–C). PPARGC1A, PPARG, PLIN1, LPL, ABHD5, lncRNA PART1, lncRNA ENTPD3-AS1 in trunk keloid and DGAT2 in ear keloid showed decreased trend under paired comparation (Figures 4D–F).

## Discussion

Scars are an unavoidable product of human wound repair (9). Excessive proliferation of fibroblasts and deposition of collagen in the extracellular matrix are the basic characteristics of pathological scars (4). Colored races and people between 10 and 30 years of age are the most susceptible to keloids (9), and people at high risk of keloids are obliged to pay more attention to pathological scars when undergoing and after invasive procedures such as injections and surgery and to control inflammatory conditions that may lead to scarring, such as acne (1, 28). Given these concerns there is some urgency around the clinical translation of the basic research around keloid production to produce new combinatorial strategies to prevent, manage, and treat these pathological scars. This has led to the widespread use of tension-reducing sutures (1) and botulinum toxin-based treatments to reduce keloids resulting from wound tension (29) and accelerated the evaluation of mesenchymal and fat transplantation as promising therapies for the remediation of pathological scarring (30). Our research showed that poor lipid metabolism and adipocyte differentiation in keloids and the pre-adipocytes that may act through the processes associated with mesenchymal were decreased in the fibroblast subpopulations of keloids. Thus, we assumed that fat grafts may restore the downregulated lipo-related gene expression pattern associated with keloid development.

Fat transplantation is relatively convenient and is not only an important source of mesenchymal stem cells but other components in the graft also play an important role in the prevention and treatment of scars (16, 17). These components include adipocytes and stromal vascular fraction, which is made up of a mixture of ADSCs, pre-adipocytes, fibroblasts, monocytes, granulocytes, macrophages, endothelial cells, and cytokines (basic fibroblast growth factor, insulin-like growth factor-1, vascular endothelial growth factor, platelet-derived growth factor, and fibroblast growth factor-2) (31, 32).

ADSCs are known to exert some immune regulatory functions, accelerate the epithelialization of wounds by secreting anti-inflammatory factors (also included in the components of fat grafts), inhibit the secretion of inflammatory mediators, promote M1 macrophage transformation to M2, and activate and repair angiogenesis (33, 34). ADSCs can downregulate the TGF-β1/Smad signaling pathway, inhibit the expression of α-smooth muscle actin (α-SMA), downregulate collagen genes, and promote the expression of decorin, thus, playing a role in resisting excessive fibrosis (35). Antioxidant factors in fat graft components can inhibit the activation of the TGF-β1/SMAD pathway by reactive oxygen species (ROS) in chronic hypoxic environments, thereby reducing collagen deposition (36). Overall, ADSCs, adipocytes, and cytokines have synergistic effects in regulating immunity and promoting normal angiogenesis and anti-fibrotic effects through fibroblasts,pre-adipocytes, macrophages, endothelial cells, etc., in wound healing, improving the quality of scars, and reducing the risk of scar hyperplasia.

Our research found that both the chondrocytic and osteogenic pathways were enriched in keloid tissues, indicating abnormal osteogenesis and cartilage production in keloid tissues and these observations were consistent with the results of multiple studies (15, 19). In addition, we found that keloid tissues experience a significant downregulation of the genes associated with lipid metabolism, and adipocyte differentiation. Moreover, comparison of our hub genes in mesenchymal and other fibroblast subgroups from keloid tissues revealed an obvious decrease in the pre-adipocyte population of keloid stem cells. Therefore, we suggest that fat transplantation may directly supplement the protein components or pre-adipocyte cells missing from these tissues, thereby playing a part in inhibiting the progression of these pathological scars. We were able to identify specific central genes associated with keloid pathogenesis using only existing publicly available data and were able to identify a key mechanism of pathogenesis and a potential explanation for therapeutic success in these tissues. Taken together, our observations open up several novel avenues of investigation and support the continued evaluation of fat grafts in the treatment of pathological scars.

Keloid disease itself is highly heterogeneous (9) making its evaluation difficult. One of the key outcomes of this study was the production of a fairly homogenous discovery dataset and a highly heterogenous set of validation data, allowing for future investigation into keloid genetics. Both the discovery datasets in this study were produced using data from African Americans (15, 18), while the GSE158395 samples were collected from the upper trunks of three women, aged 41, 47, and 54 years (18). The public clinical information of the other discovery dataset set is incomplete but is likely to be derived from the upper trunks of three adult women (15). The verification set GSE90051 was selected from seven keloid tissues from Japanese patients and the cells used in GSE175866 were from three women with unknown clinical and demographic descriptions (12, 13, 19). Although the profile of adjacent non-lesion skin of keloid patients is likely to be different from that of normal control skin from individuals without keloid tendency (18), it is favored for gene profiling as it reduces individual confounding effects (15, 19). Most of the tumor-adjacent tissues were obtained 1 cm away from the tumor using the naked eye and are usually obtained from trimming cat ears from keloid patients (19). Therefore, additional data sets will bring more objectivity and representation to these types of analyses and larger groups and sampling efforts will help to make these observations more robust in the future.

## Conclusion

This study used five GEO datasets to evaluate the underlying physiology of keloid scars. Evaluations of these datasets revealed that keloid tissues present with significant reductions in fat-associated biological processes and fat cell differentiation. Evaluation of these outcomes produced a unique ceRNA network consisting of six hub genes, four lncRNAs, and 11 miRNAs all associated with lipid metabolism. This expression pattern was then verified in database of keloid tissues, fibroblasts from keloids, and hypertrophic scars. Preliminary verification was performed in keloid of trunk and ear. Our results suggest that keloid development relies on the downregulation of lipo-related genes and pre-adipocytes in diseased tissues and may be one of the key mechanisms underlying fat grafting mediated treatment of pathological scarring. These results allowed us to provide a theoretical support for the clinical effects of fat transplants in keloid patients, not only in terms of the positive effect on wound healing to prevent pathological scars but also the possibility the fat autograft could use a paracrine mechanism to supply the missing growth factors and proteins directly or directly replenish the pre-adipocyte content in keloids. Therefore, fat graft can be a clinical choice for keloids, which supports the further evaluation of this methodology in the treatment of keloid production.

## Data Availability Statement

Publicly available datasets were analyzed in this study. This data can be found here: http://www.ncbi.nlm.nih.gov/geo/; GSE158395, GSE92566, GSE90051, GSE178562, and GSE175866.

## Ethics Statement

The waivers of informed consent of this study was approved by the Institutional Ethics Review Board of The First Affiliated Hospital of Sun Yat-Sen University.

## Author Contributions

XL, SX, and YX designed and directed the research. XL and ZZ analyzed the study data and drawings. XL and SX drafted the manuscript. YX and SX supervised the research and edited the manuscript. All authors contributed to the article and approved the submitted version.

## Funding

This work was supported by the Guangdong Basic and Applied Basic Research Foundation [grant number 2019A1515110462].

## Conflict of Interest

The authors declare that the research was conducted in the absence of any commercial or financial relationships that could be construed as a potential conflict of interest.

## Publisher's Note

All claims expressed in this article are solely those of the authors and do not necessarily represent those of their affiliated organizations, or those of the publisher, the editors and the reviewers. Any product that may be evaluated in this article, or claim that may be made by its manufacturer, is not guaranteed or endorsed by the publisher.
