# Supplementary material for: The Downregulated Lipo-Related Gene Expression Pattern in Keloid Indicates Fat Graft Is a Potential Clinical Option for Keloid
Source: Front Med (Lausanne). 2022 May 23;9:846895. doi: 10.3389/fmed.2022.846895 (PMC9168263; doi:10.3389/fmed.2022.846895)
Supplement: Supplementary file 1 [file Table_1.pdf]

Supplementary Material

Supplementary Tables

Table S1. Clinical information of samples from tissue bank.

	Sample/Control	Gender	Age (year)	Primary Factor	Basic Situation
1	Trunk keloid 1	Female	56	Surgery	Breast Cancer
2	Trunk keloid2	Female	31	Unknown	-
3	Trunk keloid 3	Male	22	Acne	-
4	Normal dermis of trunk 1	Female	55	-	Breast Cancer
5	Normal dermis of trunk 2	Male	32	-	pilonidal sinus
6	Ear keloid 1	Female	26	Ear-piercing	Family history of keloids
7	Ear keloid 2	Female	9	Incised Injury	-
8	Ear keloid 3	Female	25	Ear-piercing	-

Table S2. Comparation between fibroblasts from different tissues.

	S-N	HTS-N		K-N		HTS-S		K-S		K-HTS	
Down	0	PDK4	2.110147e-04	PDK4	1.341859e-04	PDK4	1.842949e-04	PDK4	7.465489e-05	APOE	5.064474e-07
		APCDD1	4.586779e-02	PPARGC1A	5.129977e-04	APCDD1	3.669423e-02	PPARGC1A	1.042348e-04	PPARGC1A	6.175515e-04
				ENTPD3-AS1	2.011672e-02			ENTPD3-AS1	1.162273e-02	ENTPD3-AS1	2.257786e-02
Stable	12	8		8		8		7		7	
Up	0	APOE	3.857802e-07	LEP	4.003795e-06	APOE	1.069461e-07	LEP	4.012839e-06	LEP	5.571055e-07
		LEP	1.588957e-04			LEP	1.598297e-04	POSTN	8.292566e-03	APCDD1	3.669423e-02

N for normal skin. S for normal scar. HTS for hypertrophic scar. K for keloid. The numbers in the table are p values compared between groups.
