# Supplementary material for: The Downregulated Lipo-Related Gene Expression Pattern in Keloid Indicates Fat Graft Is a Potential Clinical Option for Keloid
Source: Front Med (Lausanne). 2022 May 23;9:846895. doi: 10.3389/fmed.2022.846895 (PMC9168263; doi:10.3389/fmed.2022.846895)
Supplement: Supplementary file 2 [file Image_1.pdf]

Supplementary Material

Supplementary Figures

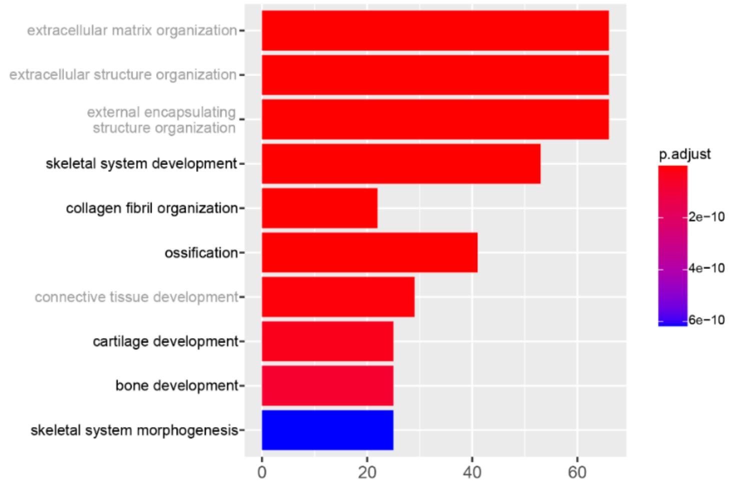

Supplementary Figure 1. Top 10 Biological Process (BP) terms describing the upregulated genes in Gene Ontology (GO) enrichment evaluations.

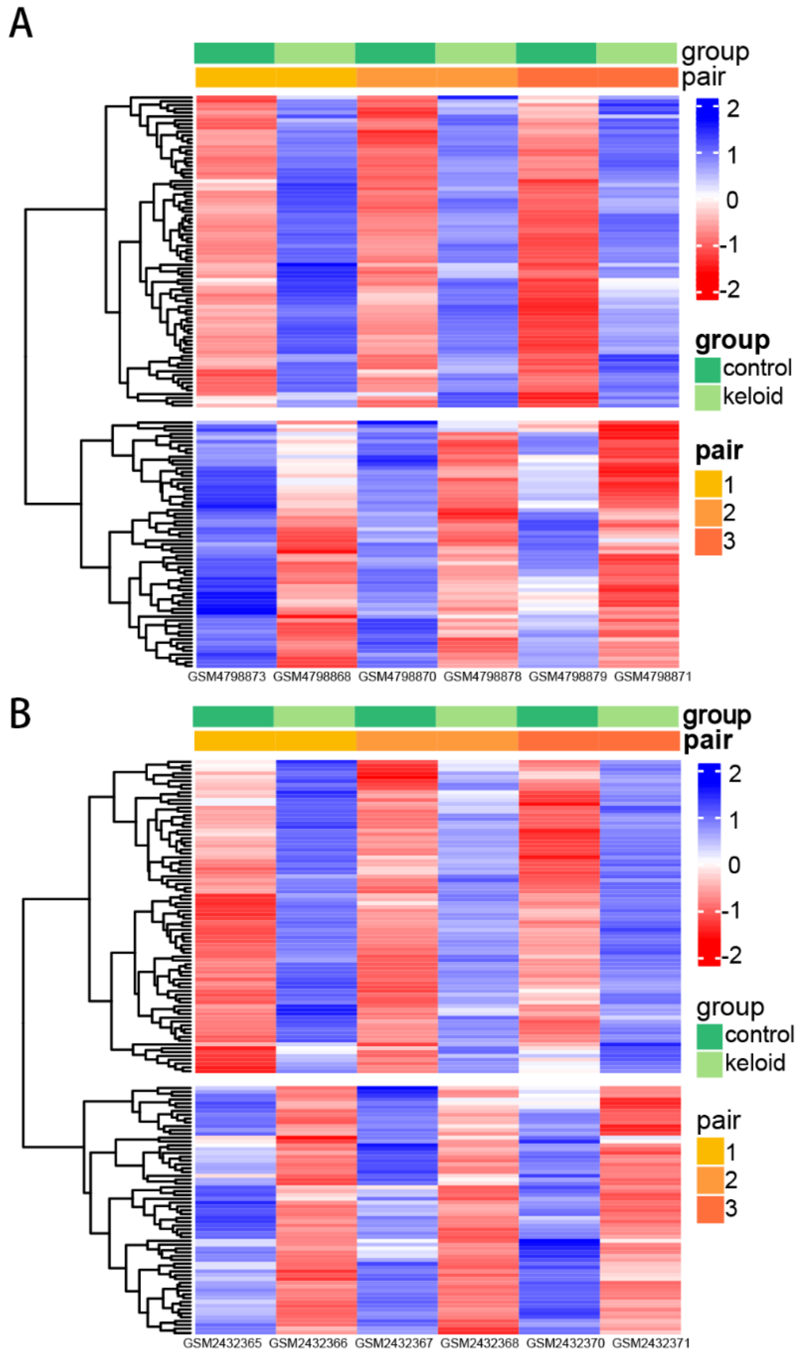

Supplementary Figure 2. Cluster heatmap of cartilage and bone genes (the upper half of each), and fat-related genes (the bottom half of each) of the top 10 Biological Process (BP) terms. A, GSE158395. B, GSE92566.

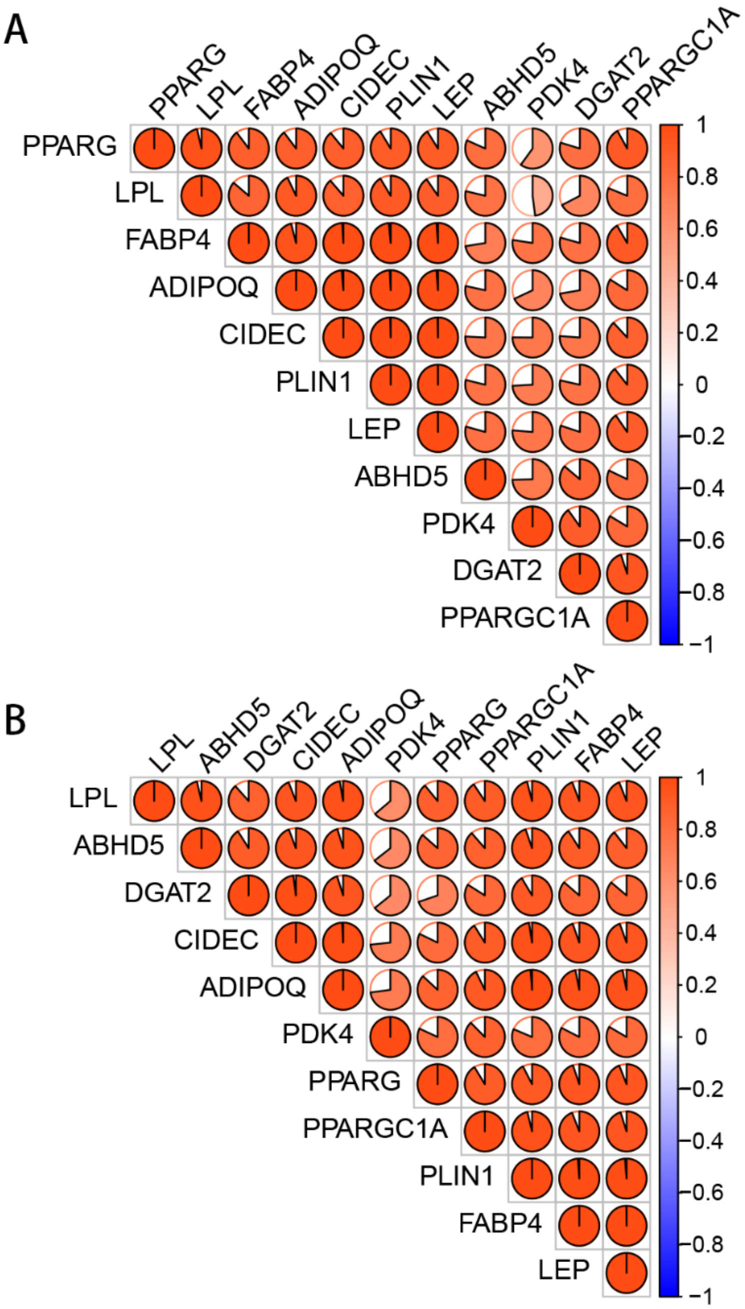

Supplementary Figure 3. Correlation heatmap of lipo-related genes of the top 10 Biological Process (BP) terms. A, GSE158395. B, GSE92566.

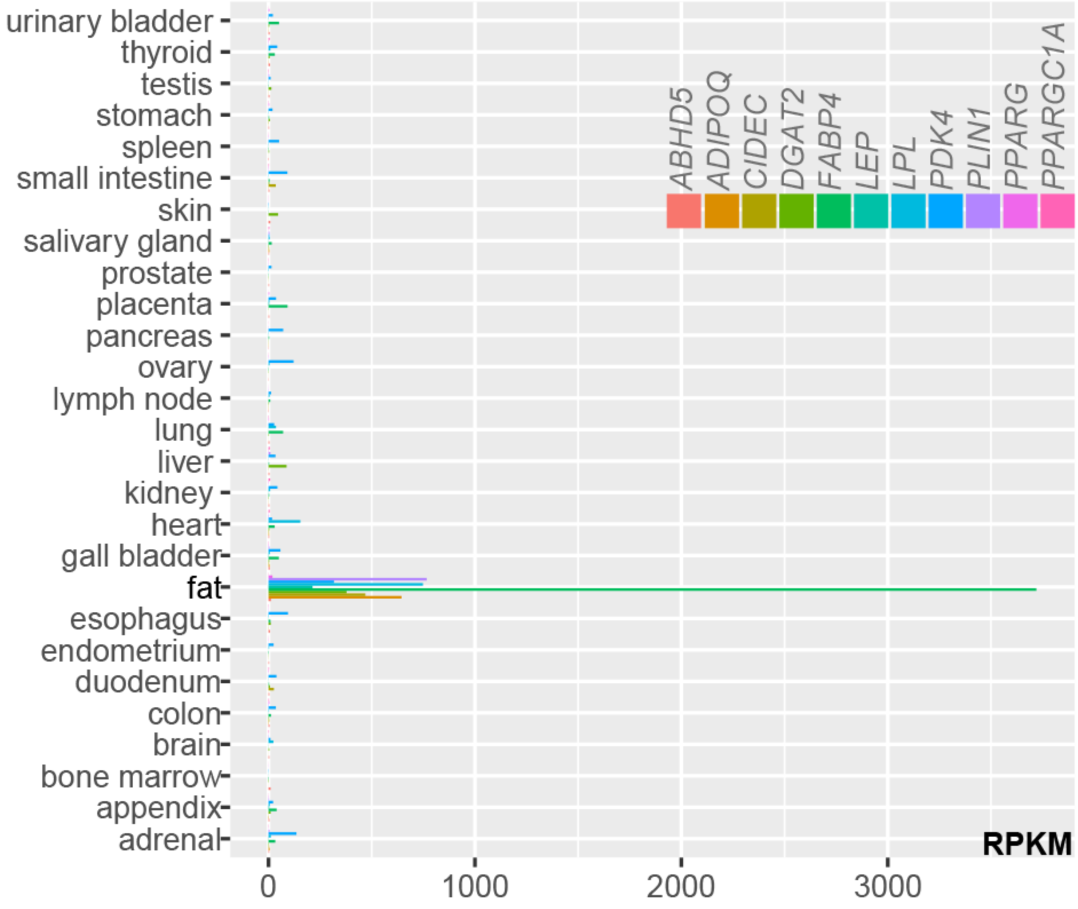

Supplementary Figure 4. Hub gene expression in normal tissues of the Gene Expression Omnibus (GEO) datasets.
